# Surgical outcomes after robot-assisted *versus* laparoscopic pancreatoduodenectomy: multicentre propensity-matched comparison from the Italian Group of Minimally Invasive Pancreatic Surgery

**DOI:** 10.1093/bjsopen/zrag152

**Published:** 2026-09-15

**Authors:** Giuseppe Quero, Roberta Menghi, Niccolò Napoli, Allegra Ripolli, Giovanni Ferrari, Michele Mazzola, Elisa Bannone, Giovanni Butturini, Massimo Giuseppe Viola, Marco Garatti, Giovanni Tebala, Fabrizio Di Benedetto, Luca Moraldi, Carlo Molino, Sergio Alfieri, Umberto Bracale, Roberto Troisi, Raffaele Dalla Valle, Marco Vivarelli, Elio Jovine, Riccardo Memeo, Gianluca Garulli, Luca Ottaviani, Stefano Berti, Andrea Coratti, Giorgio Ercolani, Roberto Salvia, Luca Morelli, Riccardo Casadei, Roberto Coppola, Alberto Brolese, Vicenzo Tondolo, Alessandro Zerbi, Ugo Boggi, the IGOMIPS Registry Study Group, A Gemini, L Tirloni, A Maffongelli, A Giani, V P Barbieri, R Ballarin, A Giardino, M Cesare Giglio, M Giuffrida, R Rossi, A Manzoni, G Zimmitti, L Mastrangelo, F Valentina, L Veneroni, G L Baiocchi, M P F Dorma, G Capretti, A Mereu, P Di Gioia, C Ricci, S Marcucci, S Tramontano, D Caputo, C Fiorillo, A Comandatore, A Esposito

**Affiliations:** Digestive Surgery Unit, Fondazione Policlinico Universitario Agostino Gemelli IRCCS di Roma, Rome, Italy; Dipartimento di Medicina e Chiurgia Traslazionale, Università Cattolica del Sacro Cuore di Roma, Rome, Italy; Digestive Surgery Unit, Fondazione Policlinico Universitario Agostino Gemelli IRCCS di Roma, Rome, Italy; Dipartimento di Medicina e Chiurgia Traslazionale, Università Cattolica del Sacro Cuore di Roma, Rome, Italy; Division of General and Transplant Surgery, University of Pisa, Pisa, Italy; Division of General and Transplant Surgery, University of Pisa, Pisa, Italy; Department of Minimally Invasive and Oncologic Surgery, ASST Grande Ospedale Metropolitano Niguarda, Milan, Italy; Department of Minimally Invasive and Oncologic Surgery, ASST Grande Ospedale Metropolitano Niguarda, Milan, Italy; Unit of HPB Surgery, Pederzoli Hospital, Peschiera del Garda, Italy; Unit of HPB Surgery, Pederzoli Hospital, Peschiera del Garda, Italy; General Surgery Department, G. Panico Hospital, Tricase, Lecce, Italy; Department of General Surgery, Fondazione Poliambulanza Istituto Ospedaliero, Brescia, Italy; Digestive and Emergency Surgery Unit, Azienda Ospedaliera S. Maria, Terni, Italy; HPB Surgery and Liver Transplant Unit, University of Modena and Reggio Emilia, Modena, Italy; HPB Surgery Unit, AOU Careggi Firenze, Firenze, Italy; Department of Oncological Surgery Team 1, ‘'Antonio Cardarelli'’ Hospital, Naples, Italy; Digestive Surgery Unit, Fondazione Policlinico Universitario Agostino Gemelli IRCCS di Roma, Rome, Italy; Dipartimento di Medicina e Chiurgia Traslazionale, Università Cattolica del Sacro Cuore di Roma, Rome, Italy; Chirurgia Generale e d’Urgenza, AOU San Giovanni e Ruggi d’Aragona, Salerno, Italy; Department of Clinical Medicine and Surgery, Hepato-Pancreato-Biliary Surgery Unit, University of Naples Federico II, Naples, Italy; Programma di Chirurgia Oncologica ad Indirizzo Epato-Bilio-Pancreatico, Azienda Ospedaliero-Universitaria di Parma, Parma, Italy; Hepatobiliary, Pancreatic and Transplantation Surgery, AUO delle Marche, Ancona, Italy; Department of Sperimental Surgery, IRCCS Azienda Ospedaliero-Universitaria di Bologna, Bologna, Italy; Unit of Hepato-Biliary and Pancreatic Surgery, ‘F. Miulli’ General Hospital, Acquaviva delle Fonti, Bari, Italy; Department of Medicine and Surgery, LUM University, Casamassima, Italy; Chirurgia Generale d’ Urgenza Laparoscopica e Robotica, Eras Certified Centre, Ospedale Infermi Rimini, Azienda Unica della Romagna, Rimini, Italy; ASST Cremona, General Surgery Unit, Cremona, Italy; Department of General Surgery, Chirurgia Generale ed Oncologica Ospedale Michele e Pietro Ferrero, Verduno, Italy; Department of General Surgery, Emergency Surgery and Gastroenterological Diseases, Misericordia Hospital, Grosseto, Italy; Department of Medical and Surgical Sciences (DIMEC), University of Bologna, Hospital Pierantoni-Morgagni Forlì, Forlì, Italy; Pancreatic Surgery Unit, Department of Engineering for Innovation Medicine (DIMI), University of Verona, Verona, Italy; Division of General Surgery, University of Pisa, Pisa, Italy; Division of Pancreatic Surgery, IRCCS, AOUBO, Department of Internal Medicine and Surgery (DIMEC), Alma Mater Studiorum, University of Bologna, Bologna, Italy; Operative Research Unit of General Surgery, Fondazione Policlinico Universitario Campus Bio-Medico, Rome, Italy; Department of General Surgery & Hepato-Pancreato-Biliary (HPB) Unit—ASUIT, Azienda Sanitaria Universitaria Integrata del Trentino, Trento, Italy; Dipartimento di Medicina e Chiurgia Traslazionale, Università Cattolica del Sacro Cuore di Roma, Rome, Italy; General Surgery Unit, Fatebenefratelli Isola Tiberina—Gemelli Isola, Rome, Italy; Pancreatic Surgery, IRCCS Humanitas Research Hospital, Milan, Italy; Division of General and Transplant Surgery, University of Pisa, Pisa, Italy

**Keywords:** intraoperative outcomes, postoperative outcomes, pancreas-related complications, minimally invasive surgery, pancreatic surgery

## Abstract

**Background:**

Minimally invasive approaches to pancreatoduodenectomy (PD) are increasingly being adopted worldwide. However, direct comparisons between laparoscopic PD (LPD) and robot-assisted PD (RPD) remain limited and inconclusive. This study aimed to compare perioperative outcomes between LPD and RPD within a national multicentre cohort.

**Methods:**

This retrospective multicentre study included patients who underwent LPD or RPD at participating Italian centres within the Italian Group of Minimally Invasive Pancreas Surgery registry between October 2019 and December 2024. Propensity score matching was performed in a 1 : 1 ratio based on age, body mass index, American Society of Anaesthesiologists score, tumour type (pancreatic ductal adenocarcinoma *versus* other histologies), receipt of neoadjuvant chemotherapy, and requirement for vascular and/or organ resection. Primary outcomes were estimated blood loss and operative time. Secondary outcomes included conversion rate, postoperative morbidity, pancreas-related complications, reoperation, length of hospital stay, histopathological features, and deaths.

**Results:**

Among 774 minimally invasive PDs, 163 (21.1%) were LPD and 611 (78.9%) were RPD. After propensity score matching , 161 LPDs were compared with 161 RPDs. RPD was associated with a longer operative time (median 506 (interquartile range (i.q.r.) 427.5–600) *versus* 500 (411.5–551) minutes; *P* = 0.030) but lower estimated blood loss (median 200 (i.q.r. 100–337.5) ml *versus* 250 (150–500) ml; *P* < 0.001). On multivariable analysis, surgical approach was not an independent predictor of operative time, whereas RPD was independently associated with lower estimated blood loss (β −0.372, 95% confidence interval −0.641 to −0.102; *P* = 0.007). Conversion to open surgery was significantly more frequent after LPD (24 of 161; 14.9%) than RPD (11 of 161; 6.8%) (*P* = 0.023). Postoperative outcomes—including major morbidity, pancreas-specific complications, reoperation, length of hospital stay, and in-hospital or 90-day deaths—were comparable between groups. Histopathological findings were also similar.

**Conclusion:**

RPD was associated with lower estimated blood loss and a reduced conversion rate compared with LPD. However, these differences were modest in absolute terms and did not translate into clinically meaningful differences in postoperative outcomes, which remained comparable between the two approaches.

## Teaser

In this multicenter propensity score-matched study, robot-assisted pancreatoduodenectomy was compared with the laparoscopic approach. Robotic surgery was associated with lower estimated blood loss and reduced conversion rates, whereas postoperative and oncological outcomes were comparable between techniques

## Introduction

Pancreatoduodenectomy (PD) is widely recognized as one of the most complex abdominal surgical procedures and remains associated with substantial postoperative morbidity and death^1,2^. Historically, PD has been performed through an open approach. However, the adoption of minimally invasive surgery has improved perioperative outcomes across several surgical procedures, including reduced blood loss, shorter length of hospital stay (LOS), and lower complication rates^3^. These advantages have stimulated growing interest in extending laparoscopic and robotic techniques to technically demanding procedures such as PD.

The first laparoscopic PD (LPD) was performed by Gagner and Pomp^4^ in 1994. Since then, numerous studies^5–10^ have evaluated the safety and feasibility of LPD compared with open PD, although with inconsistent results. Whereas several retrospective series and three randomized clinical trials suggested favourable outcomes with LPD^5–7,9,10^, the Dutch multicentre LEOPARD-2 trial^8^ was prematurely terminated because of significantly higher postoperative complication rates in the laparoscopic arm. These conflicting findings may partly reflect the inherent technical limitations of conventional laparoscopy, including restricted degrees of freedom, the fulcrum effect, and limited depth perception associated with two-dimensional vision^11^.

The robotic platform was developed to overcome these limitations while preserving the advantages of minimally invasive surgery by providing three-dimensional visualization, enhanced dexterity with seven degrees of freedom, tremor filtration, and improved ergonomics^11^. Robotic PD (RPD), first performed in 2001 and reported by Giulianotti *et al*.^12^ in 2003, has been increasingly adopted, supported by encouraging evidence from single- and multicentre studies. Nevertheless, direct comparisons between LPD and RPD remain limited. Most available comparative studies are retrospective, single-centre, and based on relatively small cohorts, resulting in inconclusive evidence^13–17^. Some authors have reported improved perioperative outcomes after RPD, including lower blood loss, fewer postoperative complications, shorter LOS, and lower readmission rates^14^. More recently, a nationwide-matched analysis from Japan^18^ further supported these observations by demonstrating improved short-term outcomes with RPD, including reduced blood loss and lower conversion rates. Conversely, other studies have reported higher rates of postoperative pancreatic fistula (POPF), surgical site infection, and delayed gastric emptying (DGE) after RPD than after LPD^13^.

Given the evolving role of minimally invasive surgery in PD, a direct comparison between LPD and RPD is warranted to define better the relative contribution of the surgical platform to perioperative outcomes.

The Italian Group of Minimally Invasive Pancreas Surgery (IGoMIPS), established in 2019, was created to collect nationwide data prospectively from minimally invasive pancreatic centres and monitor related outcomes. Within this framework, the present multicentre observational cohort study aimed to compare intraoperative outcomes between LPD and RPD using data from the IGoMIPS registry. To minimize potential selection bias, propensity score matching (PSM) was performed using covariables expected to influence perioperative outcomes.

## Methods

### Study design and data collection

The IGoMIPS registry is a prospective registry approved by the Ethics Committee (Humanitas Research Hospital; Prot. No. Ce Humanitas ex D.M. 8/2/2013–213/19)^19^ and registered (registration number: NCT06381362 (http://www.clinicaltrials.gov)). The present study was conducted and reported in accordance with the STROBE guidelines for observational studies.

All patients who underwent elective minimally invasive PD (LPD or RPD) for any indication were retrospectively retrieved from the IGoMIPS registry and included in the present analysis. The registry prospectively includes all minimally invasive pancreatic procedures performed across 42 Italian pancreatic referral centres from October 2019 onwards, with postoperative follow-up up to 90 days after surgery. Hybrid or hand-assisted procedures were excluded to ensure procedural consistency. Surgical approaches (laparoscopic, robotic, and hybrid) were defined according to the Brescia guidelines for minimally invasive pancreatic surgery^20^. Patients scheduled for PD who ultimately underwent a different surgical procedure were excluded. To ensure the methodological robustness of the PSM analysis, only patients with complete data for the selected matching covariables were included. Patients with missing data for the primary or predefined secondary outcomes, or for clinically relevant perioperative variables required for adjusted analyses, were also excluded.

Baseline characteristics included age, sex, body mass index (BMI, kg/m^2^), American Society of Anesthesiologists (ASA) score, history of abdominal surgery, neoadjuvant therapy (NAT), and lesion type.

Surgical procedures were performed according to the standard techniques adopted at each participating centre. Operative variables included operative time, estimated blood loss (EBL), associated vascular and/or organ resection, pancreatic texture (soft *versus* firm), main pancreatic duct diameter (≤ 3 mm *versus* > 3 mm), type of PD (pylorus-preserving or classical Whipple), intraoperative pancreatic stent placement, and type of reconstruction (pancreatojejunostomy or pancreatogastrostomy).

Postoperative management followed institutional protocols. Postoperative variables included complications graded according to the Clavien–Dindo classification, POPF, post-pancreatectomy haemorrhage (PPH), DGE, bile leak, gastrojejunostomy leak, chyle leak, intra-abdominal collection, surgical site infection, sepsis, reoperation, LOS, readmission, in-hospital deaths, and 90-day deaths. POPF, PPH, and DGE were defined and graded according to the International Study Group of Pancreatic Surgery^1,21,22^.

Oncological outcomes included histopathological diagnosis, tumour diameter, number of harvested and metastatic lymph nodes, resection margin status (R) according to the Royal College of Pathologists^23^, and tumour stage according to the American Joint Committee on Cancer/Union for International Cancer Control 8th edition^24^.

### Study outcomes

The primary outcome was the comparison of intraoperative outcomes—EBL and operative time—between RPD and LPD. These endpoints were selected to reflect intraoperative performance and to allow a standardized comparison between surgical approaches across multiple centres.

Secondary outcomes included conversion to open surgery, postoperative morbidity, pancreas-related complications, postoperative transfusion, reoperation, LOS, histopathological features, and deaths.

To account for centre-level clustering and temporal trends related to the progressive adoption of RPD, sensitivity analyses were performed for the primary outcome (EBL) and key secondary outcomes (conversion to open surgery), with additional exploratory analyses for failure-to-rescue, using mixed-effects regression models including centre as a random intercept, and calendar era (2019–2020, 2021–2022, and 2023–2024) as a fixed-effect covariable.

### Statistical analysis

To reduce selection bias between the RPD and LPD cohorts, PSM was performed in a 1 : 1 ratio. Propensity scores were estimated using a multivariable logistic regression model including age, BMI, ASA score, tumour histology (pancreatic ductal adenocarcinoma *versus* other histologies), NAT, and associated vascular and/or organ resection as covariables, selected based on their potential influence on the primary outcomes. Patients were matched using nearest-neighbour matching without replacement and a caliper width of 0.2 standard deviations of the logit of the propensity score.

Balance between groups was assessed using standardized mean differences (MDs), with values < 0.1 considered indicative of adequate balance.

Continuous variables are reported as median (interquartile range (i.q.r.)), whereas categorical variables are reported as number and percentages. Univariable analysis was performed using Student’s *t* tests or Mann–Whitney *U* tests for continuous variables, and Fisher’s tests or χ2 tests for categorical variables, as appropriate. For all analyses, a two-sided *P* value < 0.05 was considered statistically significant.

Univariable and multivariable analyses were performed to explore variables associated with operative time and EBL. Both outcomes were analysed as continuous variables. Operative time was analysed using linear regression, whereas EBL was log-transformed to account for its right-skewed distribution and improve model fit. Given the exploratory aim of the analysis, both baseline and intraoperative variables were included. Results are reported as unstandardized regression coefficients (β) with corresponding 95% confidence intervals (c.i.) and *P* values. A two-sided *P* value < 0.05 was considered statistically significant. All analyses were performed using IBM SPSS Statistics® version 25 (IBM Corp., Armonk, NY, United States).

To explore the potential impact of missing data, baseline characteristics of included and excluded patients were compared in the overall study population.

Sensitivity analyses were performed using mixed-effects regression models including centre as a random intercept and calendar era as a fixed-effect covariable. A linear mixed-effects model was applied for the continuous outcome of EBL, whereas mixed-effects logistic regression models were used for the binary outcomes of conversion to open surgery and failure-to-rescue after major postoperative complications.

## Results

### Before matching

#### Clinico-demographic characteristics

**Figure 1:**
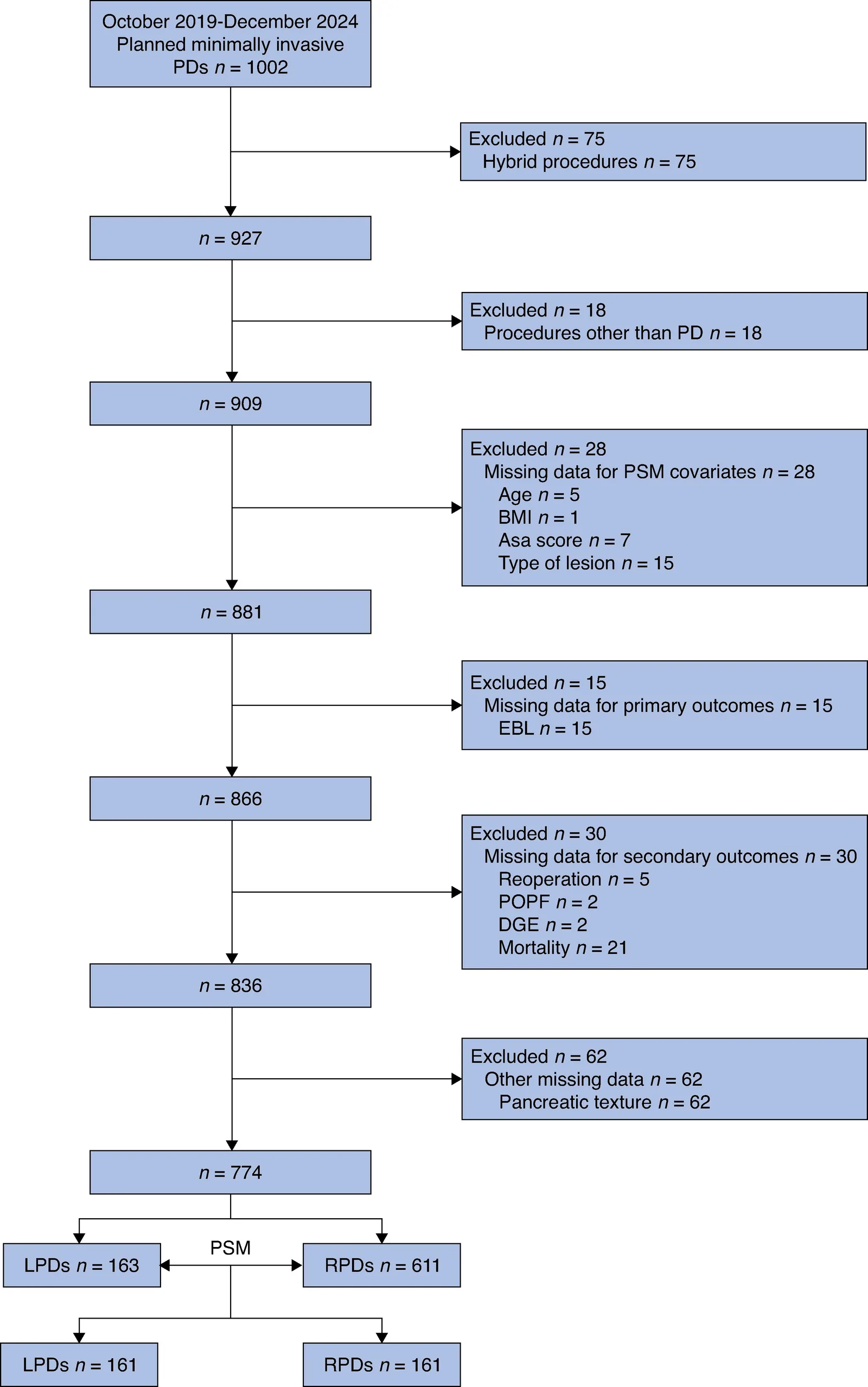
Patient selection flowchart After applying predefined inclusion and exclusion criteria to 1002 planned minimally invasive PDs in the Italian Group of Minimally Invasive Pancreas Surgery registry, 774 patients were included in the final cohort. Distribution of LPD and RPD before and after PSM is reported. PD, pancreatoduodenectomy; LPD, laparoscopic PD; RPD, robotic PD, PSM, propensity score matching, BMI, body mass index, ASA, American Society of Anesthesiologists; EBL, estimated blood loss; POPF, postoperative pancreatic fistula; DGE, delayed gastric emptying.

**Figure 2:**
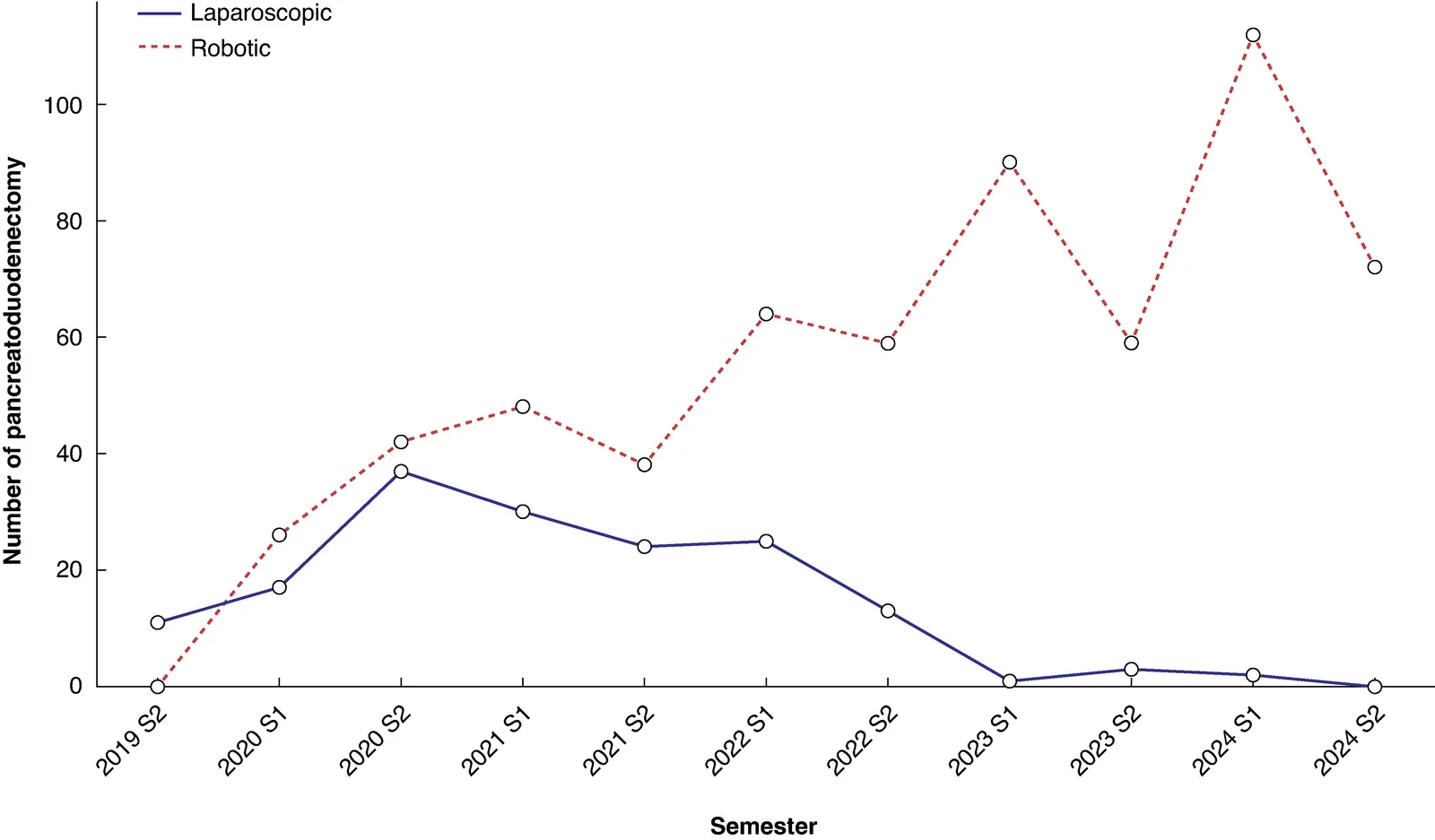
Line graph depicting the temporal distribution of laparoscopic and robotic pancreatoduodenectomy across consecutive semesters S1: January–June; S2: July–December, from October 2019 to December 2024.

Between October 2019 and December 2024, a total of 1002 planned minimally invasive PDs were recorded in the IGoMIPS registry. After applying the predefined inclusion and exclusion criteria, 774 patients (77.2%) were included in the final analysis. Among these, 163 (21.1%) underwent LPD and 611 (78.9%) underwent RPD (*Fig. 1*). To explore the potential impact of selection bias related to missing data, baseline characteristics of included and excluded patients were compared (*Table S1*). Baseline characteristics were comparable across all evaluated variables. Over the study period, a clear temporal shift in surgical approach was observed, with a progressive decline in the laparoscopic approach and a concomitant increase in the robotic approach, which became predominant in the most recent semesters (*Fig. 2*). Before PSM, patients in the LPD cohort were significantly older than those in the RPD group (median 71 (i.q.r. 62–77) *versus* 68 (59–75) years; *P* = 0.005). All other clinico-demographic characteristics, including BMI, ASA score, previous abdominal surgery, and receipt of NAT, were comparable between the two groups (*Table 1*).

**Table 1:** Clinico-demographic characteristics of the unmatched and matched cohort

	LPD	RPD	*P*
**Unmatched cohort (*n* = 774)**	(*n* = 163)	(*n* = 611)	
Sex			
Male	95 (58.3%)	333 (54.5%)	0.383
Female	68 (41.7%)	278 (45.5%)	
Age (years), median (i.q.r.)	71 (62–77)	68 (59–75)	0.005
Body mass index, median (i.q.r.)	24.5 (22.3–27.2)	24.2 (22.1–26.7)	0.370
ASA score			
1–2	100 (61.3%)	363 (59.4%)	0.090
3–4	63 (38.7%)	248 (40.6%)	
Previous abdominal surgery	68 (42.0%)	210 (34.4%)	0.072
NAT	12 (7.4%)	39 (6.4%)	0.653
Lesion type			
PDAC	67 (41.1%)	259 (42.4%)	0.764
Other	96 (58.9%)	352 (57.6%)	
**Matched cohort (*n* = 322)**	(*n* = 161)	(*n* = 161)	
Sex			
Male	94 (58.4%)	93 (57.8%)	0.910
Female	67 (41.6%)	68 (42.2%)	
Age (years), median (i.q.r.)	71 (62.0–76.5%)	69 (60.0–75%)	0.143
Body mass index, median (i.q.r.)	24.5 (22.3–27.2)	23.7 (21.6–26.8)	0.192
ASA score			
1–2	99 (61.5%)	99 (61.5%)	0.180
3–4	62 (38.5%)	62 (38.5%)	
Previous abdominal surgery	68 (42.5%)	63 (39.1%)	0.543
NAT	12 (7.5%)	9 (5.6%)	0.502
Lesion type			
PDAC	66 (41.0%)	66 (41.0%)	1
Other	95 (59.0%)	95 (59.0%)	

Values are *n* (%) unless otherwise stated. LPD, laparoscopic pancreatoduodenectomy; RPD, robotic pancreatoduodenectomy; i.q.r., interquartile range; ASA, American Society of Anesthesiologists; NAT, neoadjuvant therapy; PDAC, pancreatic ductal adenocarcinoma.

#### Intraoperative outcomes (*Table 2*)

RPD was associated with lower EBL (median 200 (i.q.r. 100–300) ml *versus* 250 (150–500) ml; *P* < 0.001), a higher rate of pylorus-preserving procedures (73.2% (447) *versus* 14.1% (23); *P* < 0.001) and more frequent pancreatic stent placement (428 (72.5%) *versus* 91 (56.5%); *P* < 0.001). No significant differences were observed in operative time (median 500 (i.q.r. 407–552) and 495 (420–580) minutes (min) for LPD and RPD, respectively; *P* = 0.100) or type of pancreatic anastomosis, with pancreatojejunostomy representing the most common reconstruction in both cohorts (155 (95.1%) and 577 (91.1%) in the LPD and RPD groups, respectively; *P* = 0.130). Similarly, an equivalent number of vascular resections were performed in both cohorts (10 (6.1%) *versus* 37 (6.1%); *P* = 0.971). Conversely, conversion to open surgery was significantly more frequent in the LPD group (24 (14.7%)) compared with the RPD group (38 (6.2%)) (*P* < 0.001).

**Table 2:** Intraoperative outcomes for LPD and RPD before and after matching

	LPD	RPD	*P*
**Unmatched cohort (*n* = 774)**	(*n* = 163)	(*n* = 611)	
Operative time (min), median (i.q.r.)	500 (407–552)	495 (420–580)	0.100
Pancreatic texture			
Firm	98 (60.1%)	407 (66.6%)	0.121
Soft	65 (39.9%)	204 (33.4%)	
Pancreatic duct diameter (mm), median (i.q.r.)	3 (2–5)	4 (2–5)	0.070
Vascular resection	10 (6.1%)	37 (6.1%)	0.971
Associated organ resection	5 (3.1%)	14 (2.3%)	0.560
EBL (ml), median (i.q.r.)	250 (150–500)	200 (100–300)	< 0.001
Pancreatic stent	91 (56.5%)	428 (72.5%)	< 0.001
Whipple procedure	140 (86.4%)	164 (26.8%)	< 0.001
Pancreatic anastomosis			
P–J	155 (95.1%)	577 (91.1%)	0.130
P–G	7 (4.3%)	16 (2.5%)	
Other	0	17 (2.8%)	
Conversion	24 (14.7%)	38 (6.2%)	< 0.001
**Matched cohort (*n* = 322)**	(*n* = 161)	(*n* = 161)	
Operative time (min), median (i.q.r.)	500 (411.5–551)	506 (427.5–600)	0.030
Pancreatic texture			
Firm	97 (60.2%)	87 (54.0%)	0.262
Soft	64 (39.8%)	74 (46.0%)	
Pancreatic duct diameter (mm), median (i.q.r.)	3 (2–5)	4 (2–5)	0.221
Vascular resection	10 (6.2%)	9 (5.6%)	0.812
Associated organ resection	5 (3.1%)	5 (3.1%)	1
EBL (ml), median (i.q.r.)	250 (150–500)	200 (100–337.5)	< 0.001
Pancreatic stent	90 (56.6%)	112 (73.2%)	0.002
Whipple procedure	138 (86.3%)	49 (30.6%)	< 0.001
Pancreatic anastomosis			
P–J	154 (95.7%)	152 (94.4%)	0.021
P–G	7 (4.3%)	3 (1.9%)	
Other	0	6 (3.7%)	
Conversion	24 (14.9%)	11 (6.8%)	0.023

Values are *n* (%) unless otherwise stated. LPD, laparoscopic pancreatoduodenectomy; RPD, robotic pancreatoduodenectomy; min, minutes; i.q.r., interquartile range; EBL, estimated blood loss.

#### Postoperative outcomes

Rates of major complications and pancreas-related complications, including POPF, DGE, and PPH, were similar between the two cohorts. However, postoperative fluid collections were more frequent after RPD than LPD (191 (31.3%) *versus* 36 (22.1%); *P* = 0.021).

Overall, 104 patients (13.4%) required reoperation, with no difference between LPD and RPD cohorts (22 (13.5%) and 82 (13.5%), respectively; *P* = 0.994). Likewise, the choice of minimally invasive *versus* open approach for reoperation did not differ (*P* = 0.903). Postoperative transfusion rates were comparable between groups (*P* = 0.591). Comparable rates of in-hospital (3.7% (6) and 3.4% (21) for LPD and RPD, respectively; *P* = 0.883) and 90-day deaths (6.7% (11) and 5.1% (31) for LPD and RPD, respectively; *P* = 0.404) were documented in both groups.

Oncological outcomes, including tumour diameter, number of harvested lymph nodes, resection margin status, and pathological stage, did not differ between the two approaches. Detailed postoperative and histopathological outcomes are reported in *Table 3*.

**Table 3:** Postoperative and histopathological outcomes for LPD and RPD before and after matching

	Unmatched cohort (*n* = 774)			Matched cohort (*n* = 322)		
	LPD (*n* = 163)	RPD (*n* = 611)	*P*	LPD (*n* = 161)	RPD (*n* = 161)	*P*
**Clavien–Dindo**						
I–II	54 (52.9%)	221 (58.6%)	0.301	52 (52.0%)	64 (65.3%)	0.560
III–IV	48 (47.1%)	156 (41.4%)		48 (48.0%)	34 (34.7%)	
DGE	32 (19.8%)	123 (20.2%)	0.901	32 (20.0%)	24 (14.9%)	0.231
**DGE grade**						
A	14 (8.6%)	30 (4.9%)	0.231	14 (43.8%)	5 (20.8%)	0.202
B	14 (8.6%)	61 (10.0%)		14 (43.8%)	15 (62.5%)	
C	4 (2.5%)	31 (5.1%)		4 (12.5%)	4 (16.7%)	
POPF	28 (17.2%)	138 (21.8%)	0.202	28 (17.4%)	35 (21.7%)	0.322
**POPF grade**						
B	24 (42.1%)	106 (46.3%)	0.190	24 (42.1%)	27 (45.0%)	0.421
C	4 (7.0%)	32 (14.0%)		4 (7.0%)	8 (13.3%)	
PPH	29 (17.8%)	103 (16.9%)	0.771	28 (17.4%)	18 (11.2%)	0.110
**PPH grade**						
A	6 (3.7%)	16 (2.6%)	0.070	5 (17.9%)	4 (22.2%)	0.894
B	11 (6.7%)	39 (6.4%)		11 (39.3%)	6 (33.3%)	
C	12 (7.4%)	48 (7.9%)		12 (42.9%)	8 (44.4%)	
Fluid collection	36 (22.1%)	191 (31.3%)	0.021	36 (22.4%)	48 (29.8)	0.125
Bile leak	9 (5.5%)	50 (8.2%)	0.255	9 (5.6%)	9 (5.6)	1.000
Chyle leak	7 (4.3%)	25 (4.1%)	0.911	7 (4.3%)	6 (3.7)	0.782
Sepsis	20 (12.3%)	108 (17.7%)	0.090	20 (12.4%)	24 (14.9)	0.510
Postoperative transfusion	49 (30.1%)	197 (32.2%)	0.591	49 (30.4%)	43 (26.7%)	0.463
Reoperation	22 (13.5%)	82 (13.5%)	0.994	22 (13.7%)	16 (10.0%)	0.311
**Reoperation approach**						
Minimally invasive	9 (5.5%)	28 (4.6%)	0.903	9 (40.9%)	1 (6.2%)	0.040
Open	13 (8.0%)	54 (8.5%)		13 (59.1%)	15 (93.8%)	
Gastrojejunostomy leakage	5 (3.1%)	16 (2.6%)	0.750	5 (3.1%)	2 (1.2%)	0.251
Surgical site infection	10 (6.1%)	25 (4.1%)	0.271	10 (6.2%)	4 (2.5%)	0.100
Length of hospital stay (days), median (i.q.r.)	12 (8–22)	14 (9–27)	0.070	11 (8–21)	15 (8–25.5)	0.060
Readmission	26 (16.0%)	93 (15.5%)	0.881	26 (16.1%)	20 (12.6%)	0.363
Discharge with abdominal drainage	11 (6.7%)	67 (11.0%)	0.363	11 (6.8%)	18 (11.2%)	0.394
Postoperative deaths	6 (3.7%)	21 (3.4%)	0.883	6 (3.7%)	3 (1.9%)	0.310
90-day deaths	11 (6.7%)	31 (5.1%)	0.404	11 (6.8%)	6 (3.7%)	0.212
**Histopathological features**						
Tumour diameter (mm), median (i.q.r.)	25 (17.5–32)	25 (18–32)	0.451	24.5 (17.2–32)	25 (18–33.5)	0.430
Harvested lymph nodes	22 (16.5–30)	24 (17–34)	0.110	21.5 (16.2–30)	25 (17–34)	0.285
Positive lymph nodes	0 (0–2)	1 (0–3.2)	0.030	0 (0–2)	1 (0–4)	0.031
Positive resection margin	21 (14.5%)	83 (15.7%)	0.711	21 (14.6%)	29 (20.3%)	0.201
**T category**‡						
1	18 (26.9%)	54 (20.8%)	0.162	18 (27.3%)	14 (21.2%)	0.492
2	44 (65.6%)	166 (64.1%)		43 (65.1%)	42 (63.7%)	
3	4 (6.0%)	38 (14.7%)		4 (6.1%)	9 (13.6%)	
4	1 (1.5%)	1 (0.4%)		1 (1.5%)	1 (1.5%)	
**N category**‡						
0	24 (35.8%)	70 (27.0%)	0.251	24 (36.4%)	12 (18.2%)	0.060
1	24 (35.8%)	104 (40.2%)		23 (34.8%)	30 (45.4%)	
2	19 (28.4%)	85 (32.8%)		19 (28.8%)	24 (36.4%)	

Values are *n* (%) unless otherwise stated. ‡Tumour staging was evaluated only for pancreatic ductal adenocarcinoma lesions. LPD, laparoscopic pancreatoduodenectomy; RPD, robotic pancreatoduodenectomy; DGE, delayed gastric emptying; POPF, postoperative pancreatic fistula; PPH, post-pancreatectomy haemorrhage; i.q.r., interquartile range.

### PSM

PSM resulted in 161 matched pairs. After matching, covariable balance improved substantially, with all MDs < 0.1, indicating adequate balance between groups. Detailed balance diagnostics are reported in *Table S2* and *Fig. S1*.

### After matching

After PSM, 161 patients who underwent RPD were compared with 161 who underwent LPD. Clinico-demographic characteristics were comparable between the two approaches (*Table 1*).

#### Intraoperative outcomes

RPD was associated with a longer operative time (median 506 (i.q.r. 427.5–600) minutes) than LPD (500 (411.5–551) minutes) (*P* = 0.030). Conversely, EBL was higher in the LPD cohort (median 250 (i.q.r. 150–500) ml) compared with the RPD cohort (200 (100–337.5) ml) (*P* < 0.001). No differences were observed in the rate of vascular resection (6.2% (10) for LPD and 5.6% (9) for RPD; *P* = 0.812) (*Table 2*).

Pylorus-preserving PD was performed more frequently in the RPD group (69.5% (112)), whereas LPD patients more commonly underwent a classical Whipple procedure (138 (86.3%)) (*P* < 0.001). Although pancreatojejunostomy was the predominant reconstruction in both groups (154 (95.7%) and 152 (94.4%) in the LPD and RPD cohorts, respectively), pancreatogastrostomy was more frequently performed after LPD (7 (4.3%) *versus* 3 (1.9%); *P* = 0.021). Likewise, pancreatic stent placement was more common in the RPD group (112 (69.5%) *versus* 90 (56.6%); *P* = 0.002). Conversion to open surgery remained significantly more frequent in the LPD cohort (24 (4.9%)) compared with RPD (11 (6.8%); *P* = 0.023).

### Postoperative outcomes

Postoperative outcomes were comparable between the approaches (*Table 3*). No differences were observed in the rates of Clavien–Dindo grade ≥ 3 complications or pancreas-related complications. Reoperation was required in 38 patients (11.8%), with no significant difference between cohorts (22 (13.7%) and 16 (10.0%) in the LPD and RPD groups, respectively; *P* = 0.311). However, reoperation via an open approach was more frequent after RPD (15 of 16 (93.8%)) than after LPD (13 of 22 (59.1%)) (*P* = 0.040). Similarly, no significant differences in postoperative transfusion rates were observed between the two groups (*P* = 0.463). Postoperative in-hospital deaths did not differ significantly, although it was higher in the LPD cohort (6 (3.7%)) compared with the RPD group (3 (1.9%)) (*P* = 0.310). Similarly, 90-day deaths were comparable between the two approaches (11 (6.8%) and 6 (3.7%) for the LPD and RPD groups, respectively; *P* = 0.212).

Oncological outcomes, including tumour diameter (*P* = 0.430), number of harvested lymph nodes (*P* = 0.285), resection margin status (*P* = 0.201), and pathological staging (*P* = 0.492), were comparable between the two approaches (*Table 3*).

### Multivariable analysis of prognostic factors for operative time and EBL

On univariable analysis, female sex (*P* < 0.001), ASA score ≥ 3 (*P* < 0.001), and type of surgical procedure (pylorus-preserving *versus* Whipple; *P* = 0.002) were significantly associated with operative time. The robotic approach was also associated with operative time at univariable analysis (*P* = 0.041).

On multivariable analysis, female sex (β = −30.7 min, 95% c.i. −53.4 to −8.0; *P* = 0.008), ASA score (β = −44.6 min, −67.7 to −21.4; *P* < 0.001), and type of surgical procedure (β = −78.8 min, −106.2 to −51.3; *P* < 0.001) remained independently associated with operative time. In contrast, the effect of surgical approach was attenuated and was no longer statistically significant after adjustment (β = 12 min, −22.3 to 33.7; *P* = 0.140) (*Table 4*).

**Table 4:** Prognostic factors analysis for operative time

	Univariable regression analysis		Multivariable regression analysis	
	β (min)	*P*	β (min)	*P*
Age (years)	−0.7 (−1.90, 0.50)	0.241		
Female sex (*versus* male)	−43.5 (−67.80, −19.16)	< 0.001	− 30.7 (−53.40, −8.00)	0.008
Body mass index (kg/m^2^)	2.06 (−0.89, 5.03)	0.170		
ASA score (≥ 3 *versus* < 3)	−53.92 (−78.36, −29.48)	< 0.001	−44.60 (−67.70, −21.40)	< 0.001
Previous abdominal surgery	−0.55 (−25.53, 24.42)	0.962		
NAT	6.72 (−42.84, 56.28)	0.791		
Tumour type (PDAC *versus* others)	−9.72 (−34.58, 15.13)	0.440		
Surgical approach (RPD *versus* LPD)	28.10 (2.00, 54.70)	0.041	12.00 (− 22.30, 33.70)	0.140
Associated organ resection	45.98 (−24.38, 116.36)	0.204		
Vascular resection	11.10 (−40.80, 63.00)	0.671		
Pancreatic texture (firm *versus* soft)	13.34 (−11.34, 38.00)	0.280		
Pancreatic duct diameter (mm)	−4.00 (−8.70, 0.61)	0.090		
Pylorus-preserving *versus* Whipple	−38.40 (−62.90, −13.85)	0.002	−78.80 (−106.20, −51.30)	< 0.001
Conversion	−21.84 (−42.75, 19.94)	0.123		
Tumour diameter (mm)	0.08 (−0.90, 1.07)	0.861		

Results are expressed as unstandardized regression coefficients (β). Values in parentheses are 95% confidence intervals. Multivariable analyses were exploratory, and results should be interpreted as associational. min, minutes; ASA, American Society of Anesthesiologists; NAT, neoadjuvant therapy; PDAC, pancreatic ductal adenocarcinoma; RPD, robotic pancreatoduodenectomy; LPD, laparoscopic pancreatoduodenectomy.

On univariable analysis, age (*P* = 0.030), female sex (*P* < 0.001), surgical approach (*P* < 0.001), type of surgical procedure (*P* = 0.003), and conversion to open surgery (*P* = 0.010) were significantly associated with EBL.

On multivariable analysis, surgical approach remained independently associated with EBL, with robotic procedures associated with lower blood loss (β −0.372, 95% c.i. −0.641 to −0.102; *P* = 0.007). Female sex was also independently associated with lower EBL (β −0.456, −0.684 to −0.227; *P* < 0.001). In contrast, the effects of age, type of surgical procedure, and conversion to open surgery were no longer statistically significant after adjustment (*Table 5*).

**Table 5:** Prognostic factors analysis for EBL

	Univariable regression analysis		Multivariable regression analysis	
	β	*P*	Β	*P*
Age (years)	0.012 (0.001, 0.023)	0.030	0.009 (−0.002, 0.019)	0.101
Female sex (*versus* male)	−0.467 (−0.7, −0.234)	< 0.001	− 0.456 (−0.684, −0.227)	< 0.001
Body mass index (kg/m^2^)	0.027 (−0.001, 0.056)	0.061		
ASA score (≥ 3 *versus* < 3)	−0.113 (−0.355, 0.130)	0.360		
Previous abdominal surgery	−0.002 (−0.240, 0.230)	0.990		
NAT	0.310 (−0.157, 0.785)	0.191		
Tumour type (PDAC *versus* others)	−0.017 (−0.256, 0.221)	0.883		
Surgical approach (RPD *versus* LPD)	−0.450 (−0.687, −0.228)	< 0.001	−0.372 (−0.641, −0.102)	0.007
Associated organ resection	0.280 (−0.384, 0.958)	0.401		
Vascular resection	0.449 (−0.043, 0.942)	0.072		
Pancreatic texture (firm *versus* soft)	−0.066 (−0.303, 0.17)	0.581		
Pancreatic duct diameter (mm)	0.038 (−0.008, 0.084)	0.100		
Pylorus-preserving *versus* Whipple	−0.355 (−0.591, −0.119)	0.003	−0.05 (−0.325, 0.226)	0.720
Conversion	0.480 (0.109, 0.851)	0.010	0.348 (−0.092, 0.808)	0.231
Tumour diameter (mm)	−0.003 (−0.013, 0.006)	0.494		

Values in parentheses are 95% confidence intervals. EBL was log-transformed due to right-skewed distribution. Regression coefficients (β) represent relative changes in estimated blood loss. ASA, American Society of Anesthesiologists; NAT, neoadjuvant therapy; PDAC, pancreatic ductal adenocarcinoma; RPD, robotic pancreatoduodenectomy; LPD, laparoscopic pancreatoduodenectomy.

### Sensitivity analyses

Sensitivity analyses confirmed the robustness of the main findings (*Tables S3–S5*). After adjustment for centre-level clustering and calendar era, surgical approach remained independently associated with EBL and conversion to open surgery, with LPD showing a higher likelihood of conversion. Conversely, no significant association was observed between surgical approach and failure-to-rescue. Calendar era was not significantly associated with the investigated outcomes. These findings support the stability of the observed associations after accounting for institutional variability and temporal trends.

## Discussion

This national multicentre PSM analysis provides a comparative assessment of RPD and LPD performed in high-volume centres with established expertise in minimally invasive pancreatic surgery. The main findings indicate that RPD is associated with significantly lower intraoperative blood loss and a reduced conversion rate compared with LPD. Importantly, these advantages did not translate into significant differences in postoperative outcomes. Postoperative morbidity, pancreas-specific complications, and oncological outcomes were comparable between the two approaches.

The lower EBL observed after RPD is consistent with previous comparative studies^15,25–27^ reporting improved intraoperative outcomes with the robotic approach . This may be partly explained by the technical advantages of the robotic platform, including three-dimensional visualization, enhanced dexterity, tremor filtration, and improved instrument stability, which may facilitate precise dissection and haemostasis. Similarly, the lower conversion rate observed in the RPD group may reflect the ability of the robotic platform to manage technically demanding intraoperative scenarios better, as previously suggested in comparative studies^15,17,28^. These findings are also in line with more recent evidence, including a recent nationwide matched analysis from Japan^18^, which reported reduced blood loss and lower conversion rates after RPD compared with laparoscopic and open approaches. Conversely, other authors^17,28^ have reported comparable EBL between RPD and LPD but a reduced need for intraoperative transfusions in the robotic cohort. Taken together, the available evidence suggests that the intraoperative benefits of RPD are plausible, but their magnitude may vary according to patient selection, institutional expertise, and procedural standardization. In this context, the role of the learning curve should also be considered when interpreting these intraoperative findings. Both LPD and RPD are technically demanding procedures, and surgeon experience is known to influence operative performance, conversion rates, and perioperative outcomes. Several studies^14,26,28–31^ have shown that increasing experience with RPD is associated with progressive improvements in operative efficiency and short-term outcomes. In the present study, the progressive adoption of RPD over time may partly reflect increasing institutional and team proficiency with the robotic platform. Therefore, some of the observed advantages of RPD, particularly lower blood loss and fewer conversions, may be related not only to the intrinsic technical features of the robotic system but also to procedural maturity and accumulated experience. However, surgeon-specific experience metrics were not available in the registry, and this aspect could not be directly analysed. Notably, sensitivity analyses accounting for calendar era did not show a significant temporal effect on the investigated outcomes, supporting the robustness of the main findings.

Operative time remains a debated issue in minimally invasive pancreatic surgery. Previous comparative studies and meta-analyses^5,15,17,26,28,32,33^ have reported heterogeneous results, with some showing similar operative times between LPD and RPD and others suggesting longer procedures during the early phase of robotic adoption. In the present study, operative time was slightly longer in the robotic cohort, but surgical approach was not an independent determinant after adjustment. This suggests that factors other than the platform itself may play a more relevant role. Differences in procedural strategy may have contributed to this finding. In particular, RPD was more frequently performed as a pylorus-preserving procedure, which often requires hand-sewn gastrointestinal reconstruction, whereas LPD more commonly involved antrectomy and stapled reconstruction. Operative time should therefore be interpreted in the context of technical heterogeneity rather than as a direct measure of platform performance alone.

Despite differences in intraoperative outcomes, postoperative results were similar between groups. Rates of major complications and pancreas-specific complications, including POPF, DGE, and PPH, did not differ significantly between RPD and LPD. This is consistent with recent systematic reviews and network meta-analyses^11,15–17,34^ showing comparable postoperative morbidity between the two minimally invasive approaches. The similarity in pancreas-related complications may be explained by the balanced distribution of established risk factors, including pancreatic texture, duct diameter, and underlying pathology. LOS, readmission, postoperative transfusion, reoperation, and deaths were also comparable, suggesting that the modest intraoperative advantages observed with RPD did not translate into measurable short-term clinical benefits in this cohort.

The management of postoperative complications requiring reintervention deserves specific consideration. In this study, reinterventions after LPD were more frequently performed using a minimally invasive approach, whereas those after RPD were more often managed through laparotomy. This difference may reflect logistical limitations, as robotic platforms are not always readily available in urgent or emergency settings, unlike conventional laparoscopy. Although evidence on robotic surgery in emergency settings remains limited^35^, differences in reintervention modality may influence the effectiveness of surgical rescue following severe complications and could partially affect downstream outcomes. In this context, it is noteworthy that 90-day deaths, although not statistically different, were nearly doubled in the LPD cohort. This numerical disparity may be influenced by multiple factors, including patient selection and perioperative management. However, the present study was not designed to assess the relationship between reintervention strategy and deaths, and no specific analysis was performed to explore this association. Therefore, any potential link between these findings should be considered hypothesis-generating only and warrants further investigation in dedicated studies.

The higher utilization of internal stents observed in the RPD group represents an additional noteworthy finding. This difference may reflect surgeon preference or reconstructive strategy when performing robotic pancreatoenteric anastomoses, rather than an intrinsic requirement of the robotic approach. Importantly, increased stent use did not translate into differences in fistula rates, suggesting that this variable did not adversely affect clinical outcomes but should nevertheless be acknowledged as part of the technical heterogeneity inherent to multicentre analyses.

Regarding oncological adequacy, RPD and LPD yielded comparable histopathological outcomes. No relevant differences were observed in tumour diameter, lymph node harvest, resection margin status, or pathological stage. These findings are consistent with previous studies^11,17,27,29^ suggesting that minimally invasive PD can achieve oncological results comparable to conventional approaches when performed in experienced centres. Although some reports^13^ have suggested potential oncological advantages of RPD, particularly in terms of margin status and lymph node retrieval, the present study does not support a clear superiority of one minimally invasive platform over the other from an oncological standpoint.

This study has several limitations. First, its retrospective design represents the main methodological limitation. Although PSM was used to reduce baseline imbalance, residual confounding cannot be excluded. In addition, some variables known to influence postoperative outcomes, such as pancreatic texture and duct diameter, were not included in the matching model because the primary focus was on intraoperative outcomes. Second, the multicentre nature of the registry introduces variability in operative technique, perioperative management, and postoperative follow-up. Although all participating institutions were pancreatic referral centres with expertise in minimally invasive surgery, differences in surgeon experience and centre volume may still have influenced outcomes. Third, surgeon-specific learning curve data were unavailable, limiting the ability to assess directly the impact of individual experience. Fourth, selection bias related to allocation to minimally invasive surgery remains possible, as suggested by the relatively low proportion of patients receiving NAT and the limited number of vascular resections. Finally, the absence of long-term follow-up prevents assessment of oncological survival outcomes.

Despite these limitations, this study provides robust real-world evidence from a national registry including exclusively high-volume centres with substantial experience in minimally invasive pancreatic surgery. The findings support the safety and feasibility of both RPD and LPD and suggest that the robotic platform may offer specific intraoperative advantages, particularly in terms of blood loss and conversion, without compromising postoperative or histopathological outcomes.

In conclusion, RPD was associated with lower EBL and reduced conversion rates compared with LPD. However, these differences were modest and did not translate into clinically meaningful improvements in postoperative outcomes. Both approaches appear safe and feasible in experienced centres. Further prospective studies, ideally incorporating surgeon-specific learning curve metrics and long-term oncological follow-up, are warranted to define better the relative role of robotic and laparoscopic approaches in minimally invasive PD.

## Collaborators

Members of the IGOMIPS Registry Study Group: A. Gemini (Azienda Ospedaliera S. Maria, Terni, Italy), L. Tirloni (AOU Careggi, Firenze, Italy), A. Maffongelli (‘Antonio Cardarelli’ Hospital, Naples, Italy), A. Giani (ASST Grande Ospedale Metropolitano Niguarda, Milan, Italy), V. P. Barbieri (G. Panico Hospital, Tricase, Lecce, Italy), R. Ballarin (University of Modena and Reggio Emilia, Modena, Italy), A. Giardino (Pederzoli Hospital, Peschiera del Garda, Italy), M. Cesare Giglio (University of Naples Federico II, Naples, Italy), M. Giuffrida (Azienda Ospedaliero-Universitaria di Parma, Parma, Italy), R. Rossi (AUO delle Marche, Ancona, Italy), A. Manzoni (Fondazione Poliambulanza Istituto Ospedaliero, Brescia, Italy), G. Zimmitti (Fondazione Poliambulanza Istituto Ospedaliero, Brescia, Italy), L. Mastrangelo (IRCCS Azienda Ospedaliero-Universitaria di Bologna, Bologna, Italy), F. Valentina (‘F. Miulli’ General Hospital, Acquaviva delle Fonti, Bari, Italy), L. Veneroni (Ospedale Infermi, Rimini, Italy; Azienda Unica della Romagna, Rimini, Italy), G. L. Baiocchi (ASST Cremona, Cremona, Italy), M. P. F. Dorma (Misericordia Hospital, Grosseto, Italy), G. Capretti (IRCCS Humanitas Research Hospital, Milan, Italy), A. Mereu (Chirurgia Generale ed Oncologica Ospedale Michele e Pietro Ferrero, Verduno, Italy), P. Di Gioia (Hospital Pierantoni-Morgagni, Forlì, Italy), C. Ricci (University of Bologna, Bologna, Italy), S. Marcucci (Azienda Sanitaria Universitaria Integrata del Trentino, Trento, Italy), S. Tramontano (San Giovanni and Ruggi Hospital Trust, Salerno, Italy), D. Caputo (University Campus Bio-Medico di Roma, Rome, Italy; Fondazione Policlinico Universitario Campus Bio-Medico, 00128 Rome, Italy), C. Fiorillo (Fondazione Policlinico Universitario Agostino Gemelli IRCCS di Roma, Rome, Italy), A. Comandatore (University of Pisa, Pisa, Italy), A. Esposito (University of Verona, Verona, Italy).

## Supplementary Material

zrag152_Supplementary_Data

## Data Availability

The data supporting the findings of this study are available upon reasonable request from the corresponding author: atgiuseppe.quero@policlinicogemelli.it.

## References

[1] Wente MN, Veit JA, Bassi C, Dervenis C, Fingerhut A, Gouma DJ et al Postpancreatectomy hemorrhage (PPH)—an International Study Group of Pancreatic Surgery (ISGPS) definition. Surgery 2007;142:20–25 17629996 10.1016/j.surg.2007.02.001

[2] Napoli N, Kauffmann EF, Vistoli F, Amorese G, Boggi U. State of the art of robotic pancreatoduodenectomy. Updates Surg 2021;73:873–880 34014497 10.1007/s13304-021-01058-8 PMC8184559

[3] Klotz R, Mihaljevic AL, Kulu Y, Sander A, Klose C, Behnisch R et al Robotic versus open partial pancreatoduodenectomy (EUROPA): a randomised controlled stage 2b trial. Lancet Reg Health Eur 2024;39:100864 38420108 10.1016/j.lanepe.2024.100864 PMC10899052

[4] Gagner M, Pomp A. Laparoscopic pylorus-preserving pancreatoduodenectomy. Surg Endosc 1994;8:408–410 7915434 10.1007/BF00642443

[5] Wang M, Li D, Chen R, Huang X, Li J, Liu Y et al Laparoscopic versus open pancreatoduodenectomy for pancreatic or periampullary tumours: a multicentre, open-label, randomised controlled trial. Lancet Gastroenterol Hepatol 2021;6:438–447 33915091 10.1016/S2468-1253(21)00054-6

[6] Palanivelu C, Senthilnathan P, Sabnis SC, Babu NS, Srivatsan Gurumurthy S, Anand Vijai N et al Randomized clinical trial of laparoscopic versus open pancreatoduodenectomy for periampullary tumours. Br J Surg 2017;104:1443–1450 28895142 10.1002/bjs.10662

[7] Poves I, Burdío F, Morató O, Iglesias M, Radosevic A, Ilzarbe L et al Comparison of perioperative outcomes between laparoscopic and open approach for pancreatoduodenectomy: the PADULAP randomized controlled trial. Ann Surg 2018;268:731–739 30138162 10.1097/SLA.0000000000002893

[8] van Hilst J, de Rooij T, Bosscha K, Brinkman DJ, van Dieren S, Dijkgraaf MG et al Laparoscopic versus open pancreatoduodenectomy for pancreatic or periampullary tumours (LEOPARD-2): a multicentre, patient-blinded, randomised controlled phase 2/3 trial. Lancet Gastroenterol Hepatol 2019;4:199–207 30685489 10.1016/S2468-1253(19)30004-4

[9] Fall S, Souche R, Bardol T, Fabre J-M, Borie F; Montpellier-Nimes Digestive Surgery Federation. Laparoscopic versus open pancreaticoduodenectomy for pancreatic or periampullary tumors: a multicenter propensity score-matched comparative study. Surg Endosc 2025;39:3037–3048 40131468 10.1007/s00464-025-11677-6

[10] Anand U, Kodali R, Parasar K, Singh BN, Kant K, Yadav S et al Comparison of short-term outcomes of open and laparoscopic assisted pancreaticoduodenectomy for periampullary carcinoma: a propensity score-matched analysis. Ann Hepatobiliary Pancreat Surg 2024;28:220–228 38384237 10.14701/ahbps.23-144 PMC11128788

[11] Kabir T, Tan HL, Syn NL, Wu EJ, Kam JH, Goh BKP. Outcomes of laparoscopic, robotic, and open pancreatoduodenectomy: a network meta-analysis of randomized controlled trials and propensity-score matched studies. Surgery 2022;171:476–489 34454723 10.1016/j.surg.2021.07.020

[12] Giulianotti PC, Coratti A, Angelini M, Sbrana F, Cecconi S, Balestracci T et al Robotics in general surgery: personal experience in a large community hospital. Arch Surg 2003;138:777–784 12860761 10.1001/archsurg.138.7.777

[13] Emmen AMLH, Zwart MJW, Khatkov IE, Boggi U, Groot Koerkamp B, Busch OR et al Robot-assisted versus laparoscopic pancreatoduodenectomy: a pan-European multicenter propensity-matched study. Surgery 2024;175:1587–1594 38570225 10.1016/j.surg.2024.02.015

[14] DeLaura I, Sharib J, Creasy JM, Berchuck SI, Blazer DG, Lidsky ME et al Defining the learning curve for robotic pancreaticoduodenectomy for a single surgeon following experience with laparoscopic pancreaticoduodenectomy. J Robot Surg 2024;18:126 38492057 10.1007/s11701-023-01746-0

[15] Ouyang L, Zhang J, Feng Q, Zhang Z, Ma H, Zhang G. Robotic versus laparoscopic pancreaticoduodenectomy: an up-to-date system review and meta-analysis. Front Oncol 2022;12:834382 35280811 10.3389/fonc.2022.834382 PMC8914533

[16] Nassour I, Wang SC, Porembka MR, Yopp AC, Choti MA, Augustine MM et al Robotic versus laparoscopic pancreaticoduodenectomy: a NSQIP analysis. J Gastrointest Surg 2017;21:1784–1792 28819886 10.1007/s11605-017-3543-6 PMC5789456

[17] Kamarajah SK, Bundred J, Marc OS, Jiao LR, Manas D, Abu Hilal M et al Robotic versus conventional laparoscopic pancreaticoduodenectomy a systematic review and meta-analysis. Eur J Surg Oncol 2020;46:6–14 31409513 10.1016/j.ejso.2019.08.007

[18] Ikenaga N, Kumamaru H, Inomata M, Kinukawa N, Iwasaki T, Otsuka K et al Robotic versus open and laparoscopic pancreaticoduodenectomy: a nationwide matched study in Japan. Ann Surg 2025; DOI: 10.1097/SLA.0000000000006996 [Online ahead of print] 41366840

[19] Zerbi A, Capretti G, Napoli N, Belli G, Coppola R, Falconi M et al The Italian National Registry for minimally invasive pancreatic surgery: an initiative of the Italian Group of Minimally Invasive Pancreas Surgery (IGoMIPS). Updates Surg 2020;72:379–385 32468424 10.1007/s13304-020-00808-4

[20] Abu Hilal M, Van Ramshorst TME, Boggi U, Dokmak S, Edwin B, Keck T et al The Brescia internationally validated European guidelines on minimally invasive pancreatic surgery (EGUMIPS). Ann Surg 2024;279:45–57 37450702 10.1097/SLA.0000000000006006 PMC10727198

[21] Wente MN, Bassi C, Dervenis C, Fingerhut A, Gouma DJ, Izbicki JR et al Delayed gastric emptying (DGE) after pancreatic surgery: a suggested definition by the International Study Group of Pancreatic Surgery (ISGPS). Surgery 2007;142:761–768 17981197 10.1016/j.surg.2007.05.005

[22] Bassi C, Marchegiani G, Dervenis C, Sarr M, Abu Hilal M, Adham M et al The 2016 update of the International Study Group (ISGPS) definition and grading of postoperative pancreatic fistula: 11 years after. Surgery 2017;161:584–591 28040257 10.1016/j.surg.2016.11.014

[23] Campbell F, Cairns A, Duthie F, Feakins R. Dataset for Histopathological Reporting of Carcinomas of the Pancreas, Ampulla of Vater and Common Bile Duct. Version 3. London: The Royal College of Pathologists, 2019. https://www.rcpath.org/static/34910231-c106-4629-a2de9e9ae6f87ac1/G091-Dataset-for-histopathological-reporting-of-carcinomas-of-the-pancreas-ampulla-of-Vater-and-common-bile-duct.pdf (accessed 27 August 2026)

[24] Amin MB, Greene FL, Edge SB, Compton CC, Gershenwald JE, Brookland RK et al The eighth edition AJCC cancer staging manual: continuing to build a bridge from a population-based to a more “personalized” approach to cancer staging. CA Cancer J Clin 2017;67:93–99 28094848 10.3322/caac.21388

[25] Choi M, Rho SY, Kim SH, Hwang HK, Lee WJ, Kang CM. Total laparoscopic versus robotic-assisted laparoscopic pancreaticoduodenectomy: which one is better? Surg Endosc 2022;36:8959–8966 35697852 10.1007/s00464-022-09347-y

[26] Kim H, Choi SH, Jang JY, Choi M, Lee JH, Kang CM. Multicenter comparison of totally laparoscopic and totally robotic pancreaticoduodenectomy: propensity score and learning curve-matching analyses. J Hepatobiliary Pancreat Sci 2022;29:311–321 34773395 10.1002/jhbp.1078

[27] van Oosten AF, Ding D, Habib JR, Irfan A, Schmocker RK, Sereni E et al Perioperative outcomes of robotic pancreaticoduodenectomy: a propensity-matched analysis to open and laparoscopic pancreaticoduodenectomy. J Gastrointest Surg 2021;25:1795–1804 33201457 10.1007/s11605-020-04869-z

[28] Zhang X-P, Xu S, Zhao Z-M, Yu G-S, Han B, Chen X et al Outcomes of robotic versus laparoscopic pancreatoduodenectomy following learning curves of surgeons: a multicenter study on 2255 patients. Ann Surg 2023; DOI: 10.1097/SLA.0000000000006167 [Online ahead of print] 38073549

[29] Boone BA, Zenati M, Hogg ME, Steve J, Moser AJ, Bartlett DL et al Assessment of quality outcomes for robotic pancreaticoduodenectomy: identification of the learning curve. JAMA Surg 2015;150:416–422 25761143 10.1001/jamasurg.2015.17

[30] Napoli N, Kauffmann EF, Palmeri M, Miccoli M, Costa F, Vistoli F et al The learning curve in robotic pancreaticoduodenectomy. Dig Surg 2016;33:299–307 27215422 10.1159/000445015

[31] Dai M, Li P, Xu Q, Chen L, Liu W, Han X et al Learning curve of robotic pancreatoduodenectomy by a single surgeon with extensive laparoscopic pancreatoduodenectomy experience. J Robot Surg 2024;18:298 39068626 10.1007/s11701-024-02007-4

[32] Nickel F, Haney CM, Kowalewski KF, Probst P, Limen EF, Kalkum E et al Laparoscopic versus open pancreaticoduodenectomy: a systematic review and meta-analysis of randomized controlled trials. Ann Surg 2020;271:54–66 30973388 10.1097/SLA.0000000000003309

[33] Liu Q, Zhao Z, Zhang X, Wang W, Han B, Chen X et al Perioperative and oncological outcomes of robotic versus open pancreaticoduodenectomy in low-risk surgical candidates: a multicenter propensity score-matched study. Ann Surg 2023;277:e864–e871 34417366 10.1097/SLA.0000000000005160

[34] Aiolfi A, Lombardo F, Bonitta G, Danelli P, Bona D. Systematic review and updated network meta-analysis comparing open, laparoscopic, and robotic pancreaticoduodenectomy. Updates Surg 2021;73:909–922 33315230 10.1007/s13304-020-00916-1 PMC8184540

[35] Milone M, Anoldo P, de’Angelis N, Coccolini F, Khan J, Kluger Y et al The role of RObotic surgery in EMergency setting (ROEM): protocol for a multicentre, observational, prospective international study on the use of robotic platform in emergency surgery. World J Emerg Surg 2024;19:20 38835071 10.1186/s13017-024-00542-x PMC11149189

