# Supplementary material for: Surgical outcomes after robot-assisted versus laparoscopic pancreatoduodenectomy: multicentre propensity-matched comparison from the Italian Group of Minimally Invasive Pancreatic Surgery
Source: BJS Open. 2026 Sep 15;10(5):zrag152. doi: 10.1093/bjsopen/zrag152 (PMC13575687; doi:10.1093/bjsopen/zrag152)
Supplement: Supplementary file 1 [file zrag152_supplementary_data.docx]

**Robot-assisted vs laparoscopic pancreaticoduodenectomy: a multicenter propensity-matched comparison of surgical outcomes from the Italian Group of Minimally Invasive Pancreatic Surgery (IGoMIPS)**

Giuseppe Quero^1,2^, MD, PhD; Roberta Menghi^1,2^, MD; Niccolò Napoli^3^, MD; Allegra Ripolli^3^, MD; Giovanni Ferrari^4^, MD; Michele Mazzola^4^, MD; Elisa Bannone^5^, MD; Giovanni Butturini^5^, MD; Massimo Giuseppe Viola^6^, MD; Marco Garatti^7^, MD; Giovanni Tebala^8^, MD; Fabrizio Di Benedetto^9^, MD; Luca Moraldi^10^, MD; Carlo Molino^11^, MD; Sergio Alfieri^1,2^, MD; Umberto Bracale^12^, MD; Roberto Troisi^13^, MD; Raffaele Dalla Valle^14^, MD; Marco Vivarelli^15^, MD; Elio Jovine^16^, MD; Riccardo Memeo^17^, MD; Gianluca Garulli^18^, MD; Luca Ottaviani^19^, MD; Stefano Berti^20^, MD; Andrea Coratti^21^, MD; Giorgio Ercolani^22^, MD; Roberto Salvia^23^, MD; Luca Morelli^24^, MD; Riccardo Casadei^25^, MD; Roberto Coppola^26^, MD; Alberto Brolese^27^, MD; Vicenzo Tondolo^2,28^, MD; Alessandro Zerbi^29^ MD; Ugo Boggi^3^, MD and the IGOMIPS Registry Study Group

^1^ Digestive Surgery Unit, Fondazione Policlinico Universitario Agostino Gemelli IRCCS di Roma, Rome, Italy

^2^ Dipartimento di Medicina e Chiurgia Traslazionale, Università Cattolica del Sacro Cuore di Roma, Rome, Italy

^3^ Division of General and Transplant Surgery, University of Pisa, Pisa, Italy

^4^ Department of Minimally Invasive and Oncologic Surgery, ASST Grande Ospedale Metropolitano Niguarda, Milan, Italy

^5^ Unit of HPB Surgery, Pederzoli Hospital, Peschiera del Garda, Italy

^6^ General Surgery Department, G. Panico Hospital, Tricase, Lecce, Italy

^7^ Department of General Surgery, Fondazione Poliambulanza Istituto Ospedaliero, Brescia, Italy

^8^ Digestive and Emergency Surgery Unit Azienda Ospedaliera S.Maria Terni, Italy

^9^ HPB Surgery and Liver Transplant Unit, University of Modena and Reggio Emilia, Modena, Italy

^10^ HPB Surgery Unit, AOU Careggi Firenze, Italy

^11^ Department of Oncological Surgery Team 1, ''Antonio Cardarelli'' Hospital, Naples, Italy

^12^ Chirurgia Generale e d’Urgenza, AOU San Giovanni e Ruggi d’Aragona, Salerno, Italy

^13^ Department of Clinical Medicine and Surgery, Hepato-Pancreato-Biliary Surgery Unit, University of Naples Federico II, Naples, Italy

^14^ Programma di Chirurgia Oncologica ad indirizzo epato-bilio-pancreatico, Azienda Ospedaliero-Universitaria di Parma, Parma, Italy

^15^ Hepatobiliary, Pancreatic and Transplantation Surgery, AUO delle Marche, Ancona

^16^ IRCCS Azienda Ospedaliero-Universitaria di Bologna

^17^ Unit of Hepato-Biliary and Pancreatic Surgery, "F. Miulli" General Hospital, Acquaviva delle Fonti, 70021 Bari, Italy. Department of Medicine and Surgery, LUM University, 70010 Casamassima, Italy.

^18^ Chirurgia Generale d' urgenza Laparoscopica e Robotica;Eras certified Centre; Ospedale Infermi Rimini; Azienda Unica della Romagna

^19^ ASST Cremona, General Surgery Unit, Cremona, Italy

^20^ Chirurgia Generale ed Oncologica Ospedale Michele e Pietro Ferrero, Verduno, Italy

^21^ Department of General Surgery, Emergency Surgery and Gastroenterological Diseases, Misericordia Hospital, Grosseto, Italy

^22^ Department of medical and surgical sciences (DIMEC), Univeristy of Bologna; Hospital Pierantoni-Morgagni Forlì, Italy

^23^ Pancreatic Surgery Unit, Department of Engineering for Innovation Medicine (DIMI), University of Verona, Verona, Italy

^24^ Division of General Surgery, University of Pisa, Pisa, Italy

^25^ Division of Pancreatic Surgery, IRCCS, AOUBO, Department of Internal Medicine and Surgery (DIMEC), Alma Mater Studiorum, University of Bologna, Italy

^26^ Operative Research Unit of General Surgery, Fondazione Policlinico Universitario Campus Bio-Medico, 00128 Rome

^27^ Department of General Surgery & Hepato-Pancreato-Biliary (HPB) Unit - ASUIT, Azienda Sanitaria Universitaria Integrata del Trentino, Trento, Italy

^28^ General Surgery Unit, Fatebenefratelli Isola Tiberina – Gemelli Isola, Via di Ponte Quattro Capi, 39, 00186 – Rome, Italy

^29^ Pancreatic Surgery, IRCCS Humanitas Research Hospital, Milan, Italy

**Collaborators (IGOMIPS Registry Study Group)**

Alessandro Gemini, MD: Digestive and Emergency Surgery Unit Azienda Ospedaliera S.Maria Terni, Italy

Luca Tirloni, MD: HPB Surgery Unit, AOU Careggi Firenze, Italy

Angela Maffongelli, MD: Department of Oncological Surgery Team 1, ''Antonio Cardarelli'' Hospital, Naples, Italy

Alessandro Giani, MD: Department of Minimally Invasive and Oncologic Surgery, ASST Grande Ospedale Metropolitano Niguarda, Milan, Italy

Vittoria Pia Barbieri, MD: General Surgery Department, G. Panico Hospital, Tricase, Lecce, Italy

Roberto Ballarin, MD: HPB Surgery and Liver Transplant Unit, University of Modena and Reggio Emilia, Modena, Italy

Alessandro Giardino, MD: Unit of HPB Surgery, Pederzoli Hospital, Peschiera del Garda, Italy

Mariano Cesare Giglio, MD: Department of Clinical Medicine and Surgery, Hepato-Pancreato-Biliary Surgery Unit, University of Naples Federico II, Naples, Italy

Mario Giuffrida, MD: Programma di Chirurgia Oncologica ad indirizzo epato-bilio-pancreatico, Azienda Ospedaliero-Universitaria di Parma, Parma, Italy

Roberta Rossi, MD: Hepatobiliary, Pancreatic and Transplantation Surgery, AUO delle Marche, Ancona, Italy

Alberto Manzoni, MD: Department of General Surgery, Fondazione Poliambulanza Istituto Ospedaliero, Brescia, Italy

Giuseppe Zimmitti, MD: Department of General Surgery, Fondazione Poliambulanza Istituto Ospedaliero, Brescia, Italy

Laura Mastrangelo, MD: IRCCS Azienda Ospedaliero-Universitaria di Bologna

Ferraro Valentina, MD: Unit of Hepato-Biliary and Pancreatic Surgery, "F. Miulli" General Hospital, Acquaviva delle Fonti, 70021 Bari, Italy.

Luigi Veneroni, MD: Chirurgia Generale d' urgenza Laparoscopica e Robotica; Eras certified Centre; Ospedale Infermi Rimini; Azienda Unica della Romagna

Gian Luca Baiocchi, MD: ASST Cremona, General Surgery Unit, Cremona, Italy

Maria Pia Federica Dorma, MD: Department of General Surgery, Emergency Surgery and Gastroenterological Diseases, Misericordia Hospital, Grosseto, Italy

Giovanni Capretti, MD: Pancreatic Surgery, IRCCS Humanitas Research Hospital, Milan, Italy

Alessandro Mereu, MD: Chirurgia Generale ed Oncologica Ospedale Michele e Pietro Ferrero, Verduno, Italy

Paolo Di Gioia, MD: Hospital Pierantoni-Morgagni Forlì

Claudio Ricci, MD: Division of Pancreatic Surgery, IRCCS, AOUBO, Department of Internal Medicine and Surgery (DIMEC), Alma Mater Studiorum, University of Bologna, Italy

Stefano Marcucci, MD: Department of General Surgery & Hepato-Pancreato-Biliary (HPB) Unit - ASUIT, Azienda Sanitaria Universitaria Integrata del Trentino, Trento, Italy

Salvatore Tramontano, MD: General and Emergency Surgical Unit, San Giovanni and Ruggi Hospital Trust, Salerno

Damiano Caputo, MD: Research Unit of General Surgery, Department of Medicine and Surgery, University Campus Bio-Medico di Roma, Rome, Italy; Operative Research Unit of General Surgery, Fondazione Policlinico Universitario Campus Bio-Medico, 00128 Rome

Claudio Fiorillo, MD: Digestive Surgery Unit, Fondazione Policlinico Universitario Agostino Gemelli IRCCS di Roma, Rome, Italy

Annalisa Comandatore, MD: Division of General Surgery, University of Pisa, Pisa, Italy

Alessandro Esposito, MD: Pancreatic Surgery Unit, Department of Engineering for Innovation Medicine (DIMI), University of Verona, Verona, Italy

**Corresponding author:**

Giuseppe Quero

Pancreatic Surgery Unit

Department of Surgery, Fondazione Policlinico Universitario “Agostino Gemelli”, IRCCS

Largo Agostino Gemelli, 8

00168 Rome, Italy

tel.: +39 06 30 15 51 33

fax: +39 06 30 15 65 20

ORCID ID: https://orcid.org/0000-0002-0001-9479

e-mail : giuseppe.quero@policlinicogemelli.it

**Supplementary Materials - Index**

| **Supplementary Figures and Tables** |  |
| --- | --- |
| Table S1 | *pag. 6* |
| Table S2 | *pag. 7* |
| Figure S1 | *pag. 8* |
| Table S3 | *pag. 8* |
| Table S4 | *pag. 9* |
| Table S5 | *pag. 9* |
|  |  |

**Supplementary Figures and Tables**

| **Table S1. Comparison of baseline characteristics between included and excluded patients** | | | |
| --- | --- | --- | --- |
| **Characteristic** | **Included in the analysis**  **(N = 774)** | **Excluded from the analysis**  **(N = 228)** | **p value** |
| **Age, years** | 69 (60-75) | 67 (59–74) | 0.201 |
| **Male sex, n (%)** | 428 (55.3) | 128 (56.1) | 0.820 |
| **BMI, kg/m²** | 24.2 (22.2–26.9) | 24.7 (22.1–27.4) | 0.812 |
| **ASA score, n (%)** |  |  |  |
| 1–2 | 463 (59.8) | 134 (60.6) | 0.894 |
| 3–4 | 311 (40.2) | 87 (39.4) |  |
| **Previous abdominal surgery, n (%)** | 278 (35.9) | 89 (39.0) | 0.604 |
| **NAT, n (%)** | 51 (6.6) | 18 (7.9) | 0.544 |
| **Type of lesion, n (%)** |  |  |  |
| PDAC | 326 (42.1) | 88 (41.3) | 0.830 |
| Other | 448 (57.9) | 125 (58.7) |  |

*Continuous variables are reported as median (interquartile range, IQR) and compared using the Mann–Whitney U test. Categorical variables are reported as number (percentage) and compared using the χ² test or Fisher’s exact test, as appropriate. Comparisons were performed using available data for each variable. Patients with missing data for a given variable were excluded from that specific comparison; therefore, the number of observations may vary across variables.*

| **Table S2. Standardized mean differences before and after propensity score matching** | | | |
| --- | --- | --- | --- |
| **Unmatched cohort (n:774)** | | | |
|  | **LPD (n:163)** | **RPD (n:611)** | **SMD** |
| **Baseline features** |  |  |  |
| **Age, median (IQR)** | 71 (62-77) | 68 (59-75) | 0.29 |
| **BMI, median (IQR)** | 24.5 (22.3-27.2) | 24.2 (22.1-26.7) | 0.05 |
| **ASA score, n (%)** |  |  |  |
| 1-2 | 100 (61.3) | 363 (59.4) | 0.04 |
| 3-4 | 63 (38.7) | 248 (40.6) |  |
| **NAT, n (%)** | 12 (7.4) | 39 (6.4) | 0.04 |
| **Lesion type, n (%)** |  |  |  |
| PDAC | 67 (41.1) | 259 (42.4) | 0.03 |
| Other | 96 (58.9) | 352 (57.6) |  |
| **Vascular resection, n (%)** | 10 (6.1) | 37 (6.1) | 0 |
| **Associated organ resection, n (%)** | 5 (3.1) | 14 (2.3) | 0.05 |
|  |  | |  |
| **Matched cohort (n:322)** | | | |
|  | **LPD (n:161)** | **RPD (n:161)** | **SMD** |
| **Age, median (IQR)** | 71 (62-76.5) | 69 (60-75) | 0.09 |
| **BMI, median (IQR)** | 24.5 (22.3-27.2) | 23.7 (21.6-26.8) | 0.08 |
| **ASA score, n (%)** |  |  |  |
| 1-2 | 99 (61.5) | 99 (61.5) | 0 |
| 3-4 | 62 (38.5) | 62 (38.5) |  |
| **NAT, n (%)** | 12 (7.5) | 9 (5.6) | 0.08 |
| **Lesion type, n (%)** |  |  |  |
| PDAC | 66 (41.0) | 66 (41.0) | 0 |
| Other | 95 (59.0) | 95 (59.0) |  |
| **Vascular resection, n (%)** | 10 (6.2) | 9 (5.6) | 0.026 |
| **Associated organ resection, n (%)** | 5 (3.1) | 5 (3.1) | 0 |

*SMD: standardized mean difference. Values <0.1 were considered indicative of adequate balance*

***Figure S1.*** *Standardized mean differences (SMD) for covariates included in the propensity score model before and after matching. The vertical line indicates the threshold of 0.1 for adequate balance****.***

| **Table S3. Mixed-effects linear regression analysis for estimated blood loss (EBL), including centre as a random intercept and calendar era as a fixed-effect covariate. Results are presented as F statistics and p values for each variable included in the model.** | | |
| --- | --- | --- |
| **Variable** | **F** | **p-value** |
| Surgical approach (LPD vs RPD) | 12.681 | <0.001 |
| Age | 0.073 | 0.788 |
| BMI | 0.682 | 0.410 |
| ASA score ≥3 | 2.843 | 0.092 |
| NAT | 0.145 | 0.704 |
| PDAC vs other histology | 0.194 | 0.660 |
| Vascular resection | 3.813 | 0.071 |
| Associated organ resection | 0.027 | 0.870 |
| Calendar era | 4.342 | 0.059 |

| **Table S4. Mixed-effects logistic regression analysis for conversion to open surgery, including centre as a random intercept and calendar era as a fixed-effect covariate. Results are reported as odds ratio (OR) with 95% confidence intervals (CI) and p values.** | | | |
| --- | --- | --- | --- |
| **Variable** | **OR** | **95% CI** | **p-value** |
| Surgical approach (LPD vs RPD) | 2.48 | 1.12–5.51 | 0.025 |
| Age | 0.97 | 0.94–1.01 | 0.140 |
| BMI | 0.99 | 0.92–1.06 | 0.711 |
| ASA ≥3 | 1.36 | 0.64–2.88 | 0.421 |
| NAT | 0.88 | 0.38–2.02 | 0.770 |
| PDAC vs other histology | 1.19 | 0.61–2.33 | 0.614 |
| Vascular resection | 3.92 | 1.48–10.37 | 0.006 |
| Associated organ resection | 1.67 | 0.38–7.34 | 0.503 |
| Calendar era | 0.89 | 0.56–1.41 | 0.623 |

| **Table S5. Mixed-effects logistic regression analysis for failure-to-rescue after major postoperative complications*, including centre as a random intercept and calendar era as a fixed-effect covariate. Results are reported as odds ratio (OR) with 95% confidence intervals (CI) and p values.** | | | |
| --- | --- | --- | --- |
| **Variable** | **OR** | **95% CI** | **p-value** |
| Surgical approach (LPD vs RPD) | 1.42 | 0.58–3.49 | 0.443 |
| Age | 1.03 | 0.99–1.07 | 0.120 |
| BMI | 0.98 | 0.90–1.07 | 0.631 |
| ASA ≥3 | 1.88 | 0.74–4.79 | 0.185 |
| NAT | 0.94 | 0.32–2.75 | 0.911 |
| PDAC vs other histology | 1.36 | 0.55–3.36 | 0.514 |
| Vascular resection | 4.27 | 1.36–13.42 | 0.013 |
| Associated organ resection | 1.72 | 0.29–10.05 | 0.201 |
| Calendar era | 0.91 | 0.54–1.53 | 0.720 |

***Failure-to-rescue was defined as 90-day mortality occurring after major postoperative complications (Clavien–Dindo grade ≥III)**
